# Advancing LVAD Technology: Overcoming Challenges and Shaping the Future of Mechanical Circulatory Support

**DOI:** 10.3390/jcm13247813

**Published:** 2024-12-20

**Authors:** Kostiantyn Kozakov, Zdenek Provaznik, Christof Schmid, Daniele Camboni

**Affiliations:** Department of Cardiothoracic Surgery, University Medical Center Regensburg, Franz-Josef-Strauß-Allee 11, 93053 Regensburg, Germany

**Keywords:** LVAD, advanced heart failure, infection, bleeding, hemocompatibility, adverse events, right heart failure

## Abstract

Ventricular assist devices (VADs) invigorated the management of patients with advanced heart failure, providing a lifeline for patients awaiting transplantation or requiring long-term circulatory support. This article reviews recent advances in VAD technologies, focusing on key areas of progress to overcome existing challenges and the potential for future applications. The reduction or possible elimination of infection-prone components and the evolution to transcutaneous energy transfer systems are two main research fields to reach a new quality of life category for VADs patients. Miniaturization and enhanced biocompatibility have resulted in smaller, less invasive devices with a significantly reduced risk of complications and mortality. Advances in percutaneous ventricular assist devices have emerged, contributing to the creation of less invasive options with or without intracardiac pumps, and facilitating their use for both left and right ventricles. These devices have gained more and more features, including the use of artificial intelligence. Moreover, the possibility of long-term use of intracardiac pumps offers a potential bridge to transplantation, allowing ambulation and probably also long-term circulatory support. Despite considerable advances, challenges remain, particularly in terms of improving durability, reducing the risk of ischemic events, further refining materials, and more sophisticated control and synchronization between systems that adapt to changing physiological demands.

## 1. Introduction

The number of heart failure patients continues to grow steadily despite the improvements in modern treatments [1]. Moreover, the amount of patients with advanced heart failure is also increasing, regardless of significant progress in the pharmaceutical industry [2], which leads to new demands for both patients and doctors, particularly setting new challenges for its surgical treatment.

Due to limited organ availability [3], cardiac xenotransplantation, which is still in its early stages of development [4], could be one solution. However, for significant numbers of patients with contraindications to transplantation, durable mechanical circulatory support (MCS) remains an alternative that demonstrates a suitable solution or the sole treatment option in patients with an appropriate clinical risk profile (INTERMACS) [5] after assessing modifiable risk factors using modern suitable technologies (e.g., coagulation screening, CT scan of the whole body to detect malignancies, exclusion of active infections before LVAD implantation) and carrying out its appropriate management.

Generations of clinicians, engineers, and biologists have worked on the development of LVADs since the first successful implantation of an LVAD in humans by DeBakey in 1963 and the first artificial heart implant in humans by Cooley in 1969.

One of the first devices that found widespread clinical use and demonstrated a significant improvement in two-year survival and quality of life compared to optimal medical management in the Randomized Evaluation of Mechanical Assistance for the Treatment of Congestive Heart Failure (REMATCH) trial was the HeartMate XVE (HM XVE), a first-generation implantable system [6]. Nevertheless, a lot of patients have experienced serious adverse effects, including infection, bleeding, and device dysfunctions.

To minimize these problems, next-generation devices were released, namely the Jarvik 2000 VAS and HeartMate II (HMII), an axial-continuous flow system, which demonstrated substantial clinical success, with significant improvements in survival, disabling stroke, and reoperation for device repair or replacement. But a sudden increase in thrombus-related device malfunctions by HMII was the impetus for the development of third-generation devices [7]. An assumption has been made that the narrow axial flow channel of HMII is a factor contributing to thrombus formation. Third-generation magnetic levitation centrifugal-flow systems, like HeartMate 3 (HM3) and HeartWare HVAD, have become more technically feasible due to the improvement of permanent magnets with much wider blood flow channels, which can reduce the incidence of clot formation.

Only in 2022, a total of 2517 left ventricular assist devices (LVADs) were implanted in North America, 99.8% of which were fully magnetic levitation devices. The third generation of LVADs improved 1-year and 5-year survival to 86% and 64%, respectively, in comparison to former models [8].

Notwithstanding significant technological advances, there remain numerous opportunities to further improve quality of life by reducing and preventing adverse events (AEs).

The main adverse events are infection, bleeding, stroke, and right heart failure. This review is meant to offer an overview about the current options to cope with these hurdles of LVAD therapy.

In this review, we would also like to offer a glimpse into the future technologies for mechanical circulatory support.

A comprehensive narrative literature review was conducted using the medical and clinical databases PubMed/MEDLINE (left ventricular assist device (MeSH Terms)) AND (new technologies) from 2010 to 2024. In total, 85 papers with the possibility of full access were found. In addition, searches of the multidisciplinary databases Google Scholar were conducted (key words: “long-term mechanical circulatory support (dMCS)”, ‘left ventricular assist device (LVAD)’, ‘next-generation LVAD’, ‘technological advances in LVAD’ and ‘fully implantable circulatory support systems’) from 2023 to 2024—this yielded 53 reviews, including studies focused on daily clinical practice with scientifical evidence as well as papers about historical development of MCS.

The search included studies, reviews, and articles published to date that focused on the current state of the art, technological interfaces, and potential capabilities of next-generation LVAD systems.

The records were screened by two reviewers (KK and DC). Any discrepancies and disagreements were resolved by other authors (CS and ZP). Based on the best scientific practice, 55 papers were selected for review.

## 2. Infection

### 2.1. Clinical Challenges

Infection remains one of the most common AE, affecting predominantly DL (drive line) [9] and occurring in 18% to 59% of the cases [10]. The problem of DL infections has been escalated by the current monopoly of the HeartMate 3 (HM3), as the HM3 has a thicker and stiffer driveline compared to its competitors. In attempts to resolve this situation, antibiotics in combination with anticoagulants, affecting each other’s pharmacodynamics, can further aggravate the situation and lead to other AEs (thrombosis or bleeding). As a factor that promotes the patient’s status on the transplantation waiting list (HU), it also limits the possibilities of immunosuppression after transplantation (induction of immunosuppression).

### 2.2. Technological Advances

**Figure 1:**
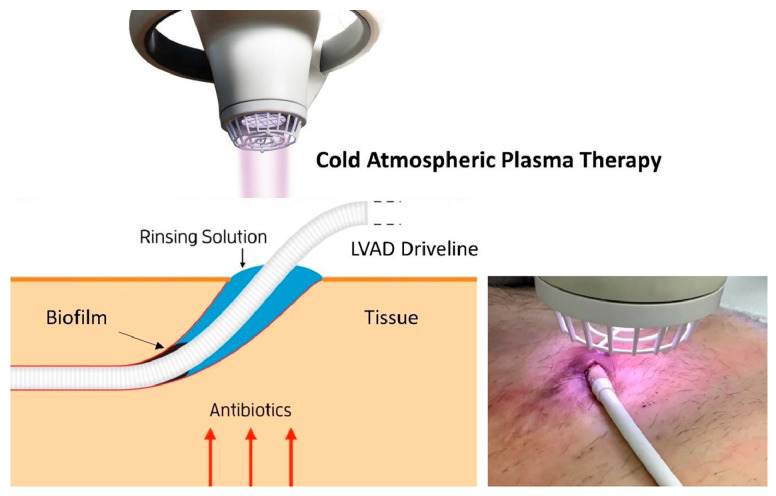
Application of cold atmospheric argongas plasma. Reprinted with permission from [14] Copyright 2023, with permission from Elsevier.

One of the modern approaches to deal with acute superficial DL infection in patients with LVAD is an application of cold atmospheric pressure argon plasma (CAP). CAP’s antimicrobial activity has shown encouraging results against a broad range of pathogens in different settings [11]. This method involves adding energy to the gas in order to create reactive oxygen and nitrogen species (ROS/RNS), which are capable of influencing the cellular oxidation and reduction balance and effectively eliminating microorganisms, including multiresistant strains [12]. The Adtec MicroPlaSter system (Adtec Healthcare, Hounslow, Middlesex, UK) for CAP application (Figure 1) is one such example. The duration of treatment ranges from 120 to 480 s depending on the depth of the wound and the stage of wound healing. Deep wound infections require a longer treatment time, as the cold plasma gas needs more time to penetrate the different layers of tissue and reach the site of biofilm formation. The CAP device is capable of treating an area of up to 12 cm^2^ per application [13]. This technique results in a decrease in the microbial burden of the patient’s wounds in 60% of cases, despite the type of causative organism, as well as a marked reduction in wound scale with a significant reduction in size, and thus demonstrates substantial cost-effectiveness in the treatment of acute superficial DL infection [13]. Despite this, the technique has not yet found broad application.

Prophylactic use of CAP demonstrated an initially signal effect of improved 30-day freedom from DLI and the results remained significant at 6 months, but after a year there was no difference in the development of DLI [15].

The use of modern DL fixation systems, such as Hollister-Platte (Hollister Incorporated, Munich, Germany), a horizontal fixation clamp for securing the DL to the abdominal wall, reduces the mobility of the DL and prevents constant trauma and inflammation, which seems to be the solution to DL infections. Using a skin adhesive like DERMABOND™, a liquid topical skin adhesive, keeps wound edges together after surgical incisions and lacerations. It provides a microbial barrier with 99% effectiveness for 72 h in vitro against those organisms mainly responsible for surgical site infections [16], and is widely used in our center.

To reduce infectious complications, investigators have focused on the development of fully implantable LVAD systems that eliminate the need for DL. The previous variants of ventricular assist devices (VADs) and total artificial hearts (TAHs) have already integrated transcutaneous energy transfer systems (TET). One of the first pumps of this kind was the AbioCor TAH. This device was used by fourteen patients in a clinical trial at five US hospitals, whereby the longest period of support was seventeen months [17]. The combination of TET and LVAD was used in the Arrow LionHeart LVD 2000 LVAD, the first fully implantable system [18]. Six patients received the device in the initial study, with a mean support duration of 245 days. The device design was substantial in size and required careful patient selection, and the internal battery can last only 20 min without external power [17].

**Figure 2:**
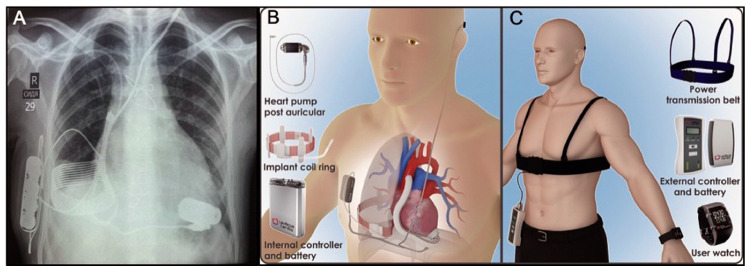
CET Leviticus FiVAD™ (LeviticusCardio, Ltd., Petach Tikva, Israel) system. (**A**) Chest X-ray illustrating the topography of the implantable components. (**B**) Implantable components. (**C**) External components. Reprinted with permission from ref. [19]. Copyright 2019, with permission from Elsevier.

Currently, the CET Leviticus FiVAD™ (LeviticusCardio, Ltd., Petach Tikva, Israel) system seems to be quite promising (Figure 2). The fully implantable VAD system with a coplanar energy transfer (CET) system is designated with two big loops with a coil-in-coil topology, which provides reliable resonance energy transfer while allowing for significant (>8 h) circulatory support supplied by an implantable battery source in combination with a continuous flow left ventricular assist device. The battery is charged wirelessly via a chest belt, and a wearable watch alerts the patient when the internal battery needs recharging, achieving a new level in quality of life [19].

## 3. Bleeding

### 3.1. Clinical Challenges

The HM3 has significantly improved hemocompatibility, with increased freedom from gastrointestinal bleeding, stroke, and device/thrombus failure due to improved coagulation stability, a fully magnetic levitated motor, artificial pulsation, and large blood flow pathways [8,20]. But bleeding remains the most common AE, requiring further research and lowering the threshold of anticoagulation. The latest trials demonstrate the ability to abandon aspirin [21], or to safely reduce the INR target range of 1.5 to 1.9 [22], and to use low molecular weight heparin in the early postoperative period, thereby allowing earlier postoperative discharge [23]. The high shear stress causing pathological von Willebrand factor degradation and impaired platelet function [24] as well as the insufficient biocompatibility [25] of the materials necessitates the use of anticoagulants and carries a significant bleeding risk.

### 3.2. Technological Advances

**Figure 3:**
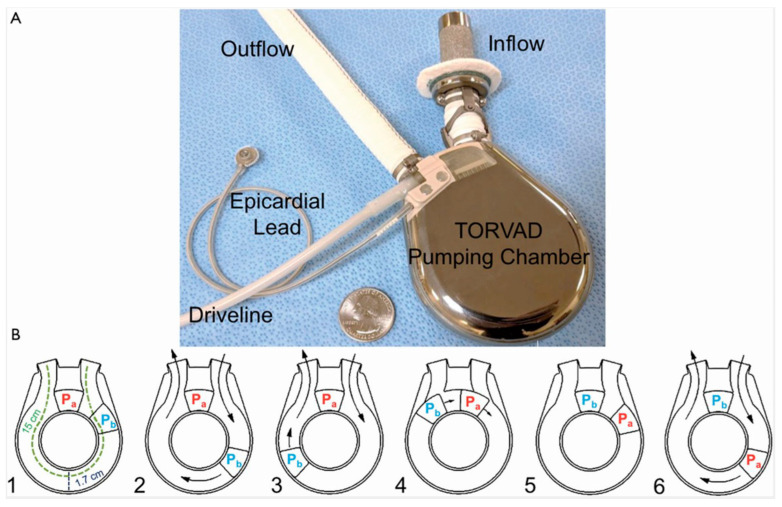
The Toroidal-Flow TORVAD Device and Mechanism of Flow (Austin, TX, USA). (**A**) The toroidal-flow TORVAD consists of an inflow cannula with sewing ring, torus pumping chamber, and outflow graft. An epicardial ECG lead senses the patient’s native heart rhythm and triggers TORVAD support with asynchronous or synchronous pulsatile, counter-pulsatile, or co-pulsatile pumping modes. (**B**) To simultaneously fill and eject, the TORVAD spins two magnetic pistons (Pa and Pb) in sequence within the doughnut-shaped torus chamber. Used with permission of AME Publishing PTE Ltd. (Austin, TX, USA), from Reinventing the displacement left ventricular assist device in the continuous-flow era: TORVAD, the first toroidal-flow left ventricular assist device by [26]; permission conveyed through Copyright Clearance Center, Inc.

One possible option for reduction in shear stress is the use of TORVAD, the toroidal-flow left ventricular assist device (Windmill Cardiovascular Systems, Inc., Austin, TX, USA, Figure 3). In contrast to CF-LVADs, which generate a peak shear stress of up to 1500 Pa (physiological level of intravascular shear stress is 2–8 Pa), the toroidal flow device provides low shear stress, minimized blood traumatization, has a pulsatile flow, and can function under physiological regulation [26]. The TORVAD’s pumping mechanism differs considerably from that of impeller-based continuous-flow left ventricular assist devices. To achieve simultaneous filling and ejection, the TORVAD employs a unique approach where one of two magnetic pistons spins within a torus-shaped chamber. While the first piston is temporarily fixated and serves as a valve between the torus’s inflow and outflow, the second piston rotates within the torus, generating a unidirectional, pulsatile blood flow pattern. The epicardial ECG lead detects the patient’s own heart rhythm and activates TORVAD support with asynchronous or synchronous pulsatile, counter-pulsatile, or co-pulsatile pump settings. The major blood flow path, at 1.7 cm, is comparable to the size of a large artery. Operating at a range of 35 to 250 RPM, the 30 mL stroke volume of the TORVAD enables it to generate approximately one to eight liters of blood flow per minute, with a peak shear stress of approximately 10 Pa [26,27].

## 4. Prevention of Thrombo-Embolic Events

### 4.1. Clinical Challenges

Despite the fact that the risk of stroke has almost halved since HM3 was implemented [28], ranging from 6 to 12.8% during the first two years [29], it still remains a significant factor leading to disability and death. Considering a 6-year evaluation period by patients with LVAD from annual INTERMACS report, neurological events represented the highest cumulative risk of death [30]. A retrospective single-center analysis by Paul Yen et al. indicates that 72.7% of perioperative strokes occur within the first 7 postoperative days, and that 86.4% of them are of ischemic origin [31]. Therefore, the risk of stroke remains a significant challenge that requires mitigation.

### 4.2. Technological Advances

A potential solution to prevent embolization is the implementation of an automatic emboli detection system for the artificial heart. Established techniques for automated embolus detection rely on the monitoring of ultrasound Doppler signals. A functional system incorporating this ultrasound Doppler technology is being developed to enable both flow assessment and embolus capture in the clinical artificial heart ReligaHeart EXT. This system will be based on an existing dual-channel multi-gate Doppler device with digital processing. A specially designed clamp-on cannula probe, equipped with 2–4 MHz piezoceramic transducers, facilitates straightforward system setup [32]. Detecting clots earlier and triggering the right therapy will likely improve clinical outcomes [25].

**Figure 4:**
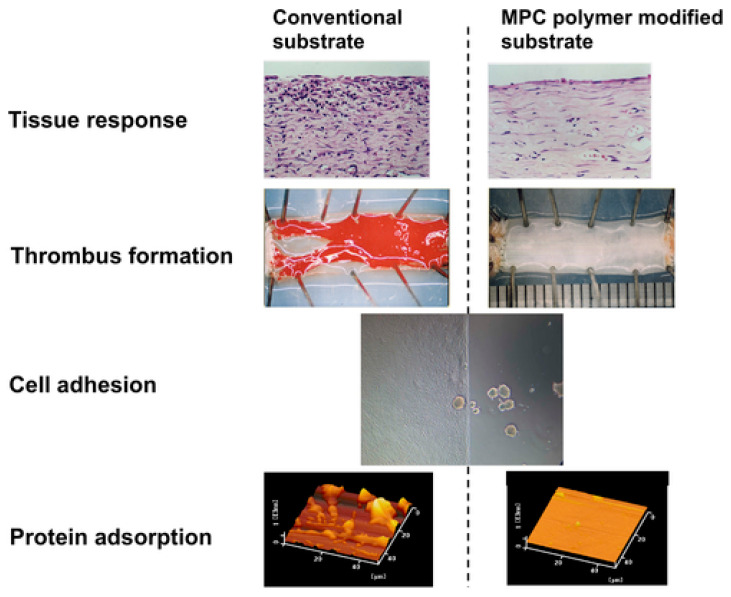
Effects of MPC polymer modification on the surface antithrombogenicity and biocompatibility. Protein adsorption was evaluated atomic force microscope after contact with protein solution for 90 min. Cell adhesion was evaluated by culturing Cos 7 kidney cells for 24 h. Thrombus formation was examined using small diameter polyester vascular grafts coated with SPU and SPU/MPC polymer blend. The vascular grafts were implanted into rabbit artery for 90 min in the SPU case and 1 month in the SPU/MPC polymer case. Tissue response was observed using SPU and SPU/MPC polymer membranes after implantation subcutaneously for 2 weeks. Tissues surrounding the specimen are stained by hematoxylin–eosin method. Reprinted with permission from ref. [34]. Copyright 2019. John Wiley & Sons Inc, the Wiley Companies.

Work with the systems’ materials demonstrates progress. Among the passive coatings, Diamond-Like Carbon (DLC) demonstrates an extremely low level of immune response and activation of coagulation factors and platelets due to its hydrophobic nature, chemical inertness, and low friction coefficient [33]. Zwitterionic films, such as phosphatidylcholine-based polymers like 2-methacryloyloxyethyl phosphorylcholine (MPC), can enhance biocompatibility by suppressing thrombin formation and platelet adhesion (Figure 4) [34].

Textured blood-contacting surfaces have shown greater biocompatibility than smooth constructs, as they promote endothelial cell adhesion and formation of a stable neointimal lining [35]. This minimizes thromboembolic risk and reduces the need for anticoagulation. Textured surfaces also increase the surface area for cell attachment and proliferation [36,37]. Examples include the textured inflow cannulas and polyurethane linings used in the HeartMate LVAD family, which also reduce the risk of device infections [36,38].

Among active coatings, the technology of Covalently bound heparin is notable. These include Carmeda BioActive Surface (CBAS) from Medtronic, which is installed on machines like the Berlin Heart LVAD, and Trillium, developed by BioInteractions Ltd. [36]. Carmeda BioActive Surface is a heparin-based coating that is covalently bound to biomaterial surfaces. This coating retains the functional activity of the immobilized heparin, allowing it to bind to the anticoagulant antithrombin in the bloodstream [39,40]. Inactive complexes formed on the heparin are then released and cleared, leaving the covalently bound heparin active and available for further inhibition [36]. Trillium is a tri-functional polymer coating for VADs that can create endothelial-like properties on the surface. It has a priming layer that binds to the blood-contacting surface, followed by a hydrophilic functional layer with covalently bound heparin. Additionally, the hydrophilic polyethylene oxide polymer in the functional layer creates an insulating water layer that resists cell adhesion and protein deposition [40].

As long as VADs require anticoagulation, the possibility of integrating the ability to measure anticoagulation parameters in real time into MCS seems to be worth consideration. Potential systems for such measurements are film bulk acoustic resonator (FBAR) for plasma and whole blood coagulation monitoring, which allows the measurement of blood coagulation properties without the use of reagents. The FBAR device has a compact size, within 1 mm^2^, reducing its form factor and manufacturing cost through batch fabrication using microelectromechanical system technology [41]. However, it remains uncertain whether this technology will be integrated into LVAD technology.

## 5. VAD Support According to Changing Physical and Therapeutic Demands

### Technological Advances

To this day, VADs provide continuous and rigid support regardless of physical need. The application and synchronization of VADs with real time pulmonary artery pressure monitoring systems, such as the CardioMEMS HF system (Abbott, San Francisco, CA, USA) [42], to allow for more precise adjustment of the LVAD and adjustment of its function to the level of physical and hence hemodynamic workload and to monitor the system’s performance could be an interesting avenue of development. It is likely that many patients with advanced heart failure and potential candidates for VADs implantation will already have such devices in the future.

Real-time evaluation of information from MCS devices and its processing by artificial intelligence (AI) has promising advantages that could lead to revolutionary changes, simultaneously performing detection and using predictive models to estimate cardiac output, ventricular workload, and to predict the development of further dynamics in the patient while adjusting therapeutic protocols and automating hemodynamic support [43,44].

## 6. Right Heart Failure

### 6.1. Clinical Challenges

Post-implantation right ventricular failure is one of the leading contributors to increased morbidity and mortality and is associated with multiple organ failure and poorer long-term survival [45]. Hence, it demands modern approaches to deal with it. Considering that afterload in patients after LVAD implantation gradually decreases, the problem of acute right ventricular failure in the early postoperative period remains particularly challenging. Despite appropriate pharmacological support and optimization of ventilation parameters, MCS for short-term right heart support is a crucial element of managing this problem.

### 6.2. Technological Advances

In addition to paracorporeal and intracorporeal devices that require surgery, there are currently two percutaneous options available for right ventricle (RV) support: Impella RP (Abiomed, Danvers, MA, USA) and TandemLife Protek Duo (TPD; TandemLife, Pittsburgh, PA, USA).

Impella RP is a system implanted percutaneously through the femoral vein under fluoroscopic control, which provides a flow of up to 4.4 liters per minute from the inferior vena cava to the pulmonary artery, unloading the right ventricle [46]. The device was safe, easy to deploy, and reliably, resulting in immediate hemodynamic benefit in patients with RV dysfunction after LVAD placement in the prospective RECOVER RIGHT study [47]. An increasing number of centers continue to use this option in appropriate patients after LVAD implantation and demonstrate relevant survival rates [48,49].

The TPD is also device of great interest for right ventricular support. It is a temporary right ventricular assist device (RVAD) that has the advantage of being placed quickly via the jugular vein, allowing ambulation in carefully monitored subjects and the use of an external ultrasonic flow probe for direct measurement of blood flow during support and can provide a flow of up to 4.5 L per minute. Unfortunately, even with the adequate TPD pump flow being reported, more than 40% of the patients in the case series died [50]. The potential benefits of this less invasive approach to right ventricular support include the avoidance of repeated sternotomies, as well as the potential to prevent the need for prolonged mechanical ventilation and costly pulmonary vasodilator therapies [50]. Further investigations are needed to select proper candidates for this option.

## 7. Intracardiac Pumps

### Technological Advances

Intracardiac pumps used as short-term support are also expanding their potential to almost mid-term MCS. Impella 5.5 (Abiomed, Danvers, MA, USA) consists of a microaxial pump positioned across the aortic valve, transferring blood from the left ventricle to the ascending aorta. Impella 5.5 with SmartAssist was approved by the Food and Drug Administration (FDA) in 2019. Implantation of the Impella 5.5 is commonly carried out through an axillary incision, allowing for continuous ambulation during cardiac recovery [51]. The device can also be inserted via direct aortic access via central cannulation and can be used in combination with other MCS devices [52]. Despite the current FDA approval of 14 days for this pump in the context of temporary MCS use as a bridge for transplantation or long-term LVAD, there are numerous reports of maintenance well above the 14-day limit without significant adverse events or mortality [51,53,54].

On the other hand, the Impella 5.5 experience demonstrated that more than a third of patients needed blood transfusions due to bleeding, leading to sensitization of patients on the waiting list for heart transplant [44,55].

Use of the aforementioned AI models can help to manage hemocompatibility issues and facilitate assessment of the probability of native heart recovery as well as early detection of potential device-related complications [44,56].

Improvement of the system toward eliminating the constant use of purge solution and upgrading to a fully implantable device with TET, as well as a fixation at the aortic valve similar to transcatheter aortic valves, would allow this device to provide more physiological blood flow compared with modern LVADs, bringing it closer to the ideal.

## 8. Conclusions

In recent years, VADs have undergone considerable changes. Innovative design approaches, new materials, and advanced monitoring systems result in significantly improved biocompatibility, reduced side effects, and physiological blood flow. These advances have opened up new opportunities on the path to improving quality of life for patients suffering from advanced heart failure.

The future of VADs looks increasingly promising, and further improvements in device design, materials, and control systems may help to solve the remaining challenges and make these life-saving technologies more available and effective for a wider range of patients.
